# Intelligent Lead: A Novel HRI Sensor for Guide Robots

**DOI:** 10.3390/s120608301

**Published:** 2012-06-14

**Authors:** Keum-Bae Cho, Beom-Hee Lee

**Affiliations:** Department of Electrical Engineering and Computer Science, Seoul National University, 1 Gwanak-ro, Gwanak-gu, Seoul 151-742, Korea; E-Mail: bhlee@snu.ac.kr

**Keywords:** guide robot, visually impaired, serial linkage, IMU, Kinect sensor, EKF

## Abstract

This paper addresses the introduction of a new Human Robot Interaction (HRI) sensor for guide robots. Guide robots for geriatric patients or the visually impaired should follow user's control command, keeping a certain desired distance allowing the user to work freely. Therefore, it is necessary to acquire control commands and a user's position on a real-time basis. We suggest a new sensor fusion system to achieve this objective and we will call this sensor the “intelligent lead”. The objective of the intelligent lead is to acquire a stable distance from the user to the robot, speed-control volume and turn-control volume, even when the robot platform with the intelligent lead is shaken on uneven ground. In this paper we explain a precise Extended Kalman Filter (EKF) procedure for this. The intelligent lead physically consists of a Kinect sensor, the serial linkage attached with eight rotary encoders, and an IMU (Inertial Measurement Unit) and their measurements are fused by the EKF. A mobile robot was designed to test the performance of the proposed sensor system. After installing the intelligent lead in the mobile robot, several tests are conducted to verify that the mobile robot with the intelligent lead is capable of achieving its goal points while maintaining the appropriate distance between the robot and the user. The results show that we can use the intelligent lead proposed in this paper as a new HRI sensor joined a joystick and a distance measure in the mobile environments such as the robot and the user are moving at the same time.

## 1. Introduction

There are approximately 285 million visually impaired people around the globe. Among them, 39 million people are totally blind. In addition, 90% of the visually handicapped live in developing countries [1]. In Korea, there are approximately 45,000 visually impaired people. However, they are provided with only 60∼70 guide dogs. Due to the aging of the population, the numbers of geriatric patients who suffer from dementia or poor physical health are on the rise. It is therefore important to develop guide robots for the elderly as well as for the visually handicapped.

A number of studies have been performed to identify a means of providing information on self location and obstacle location for the visually impaired. In an RFID-based study [2,3], a robot cart converts the location information which has been received through the RFID into voice data. As a navigation tool, various sensors are attached to clothes and then the acquired environment information with these sensors is delivered to the visually impaired through other sensory organs [4,5]. Many robots such as GuideCane [6,7], RoJi [8] and ROVI [9] have been studied in the area of autonomous robots for the visually impaired. In the above studies ultrasonic sensors are attached to the two-wheeled mobile robot as a means of detecting obstacles. The user uses the feedback force through a stick for avoiding obstacles when the steering wheel is acting. This becomes the cause that any ground bumps are directly transmitted to the user. In another study, eyeDog [10] is designed for the user to follow the robot with a dog leash. The shock to the robot is not transmitted to the user because of the flexibility of the leash, but the robot cannot acquire information about the relative location of the user and the robot.

**Figure 1.**
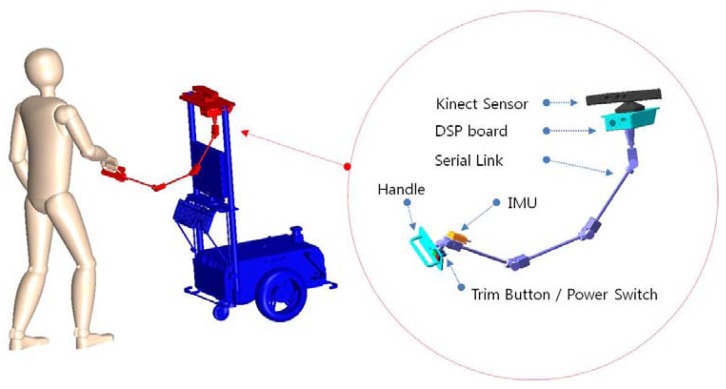
Intelligent lead and its application.

Ultimately, our goal is to fabricate a human-friendly guide robot. Among the visually-impaired population, totally blind are only 15%. For this reason human-friendly guide robot which is in a cooperative relationship with the user, not just a common mobile robot which only follows control commands is more needed. This study addresses the fabrication of a new HRI sensor (Figure 1) with which a user's location and control volumes can be acquired at the same time. We will call this an HRI sensor the “intelligent lead”. The intelligent lead proposed in this study and a 3D conceptual view about its application is shown in Figure 1.

The objective of the intelligent lead is to acquire a stable distance from the user to the robot, speed-control volume and turn-control volume, even when the robot platform with the intelligent lead is shaken on uneven ground. In this paper we explain the precise Extended Kalman Filter (EKF) procedure for this. The intelligent lead physically consists of a Kinect sensor, the serial linkage attached with eight rotary encoders, and an Inertial Measurement Unit (IMU), and their measurements are fused by the EKF. Since a user swings his/her arms back and forth while walking, it is very difficult to locate the user's body using only his/her hand position information. A new sensor is needed to locate the user's body, or one of the sensors used to detect obstacles can be used as well.

In many previous studies, an ultrasonic sensor was often used as a means for detecting obstacles. Recently, many studies on how to detect and avoid obstacles with 2D laser scanners, Kinect sensors and vision sensors [11,12] have been conducted. A vision system is light and cheap as a passive sensor. Among others, the Kinect sensor has been used to recognize a 3D object or environment in the robot industry due to its significant price competitiveness. Compared to the SR-3000 which is a commercial 3D laser scanner, it is less accurate. Since it is much cheaper, however, the Kinect sensor has been used to locate a user's body in this study.

The intelligent lead is structured to fuse the data which has been acquired through the serial linkage, Kinect and IMU sensor with the EKF. This paper is organized as follows. Section 2 describes a configuration of the major hardware of the intelligent lead. Section 3 describes how to configure the serial linkage and an IMU integrator with the EKF, and identifies a process to obtain sensor output values from the user's joint positions. Section 4 contains an analysis of the results of two tests which were been performed after attaching the proposed intelligent lead to a mobile robot. Finally, Section 5 contains conclusions and plans for the future.

## 2. Sensor System Architecture

**Figure 2.**
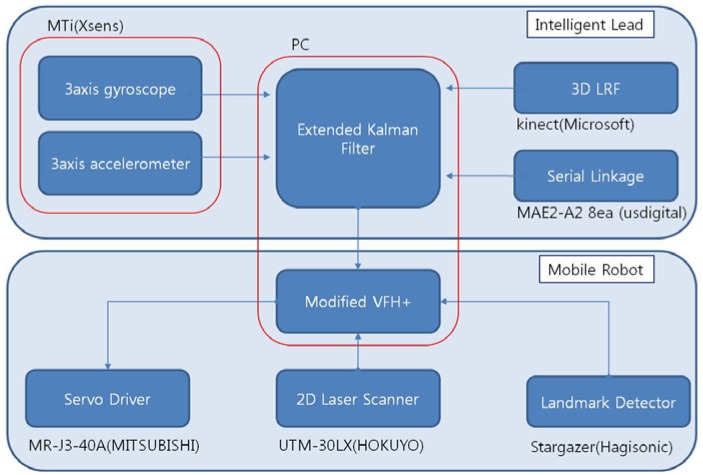
Block diagram of a proposed intelligent lead and a mobile robot for testing.

Figure 2 shows the block diagram of the intelligent lead and the mobile robot. The intelligent lead consists of an IMU, a serial linkage and the Kinect sensor.

**Figure 3.**
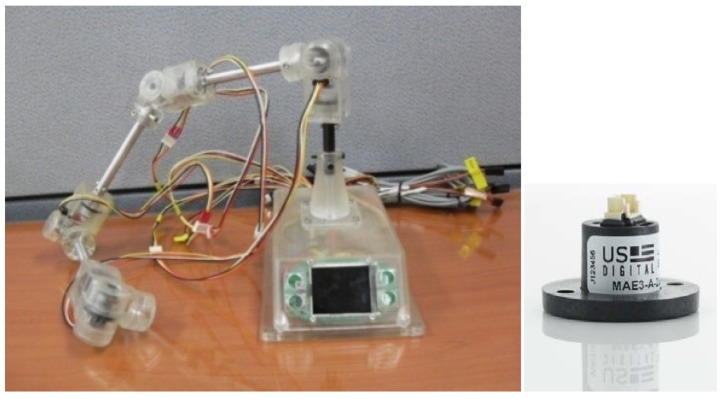
Fabricated serial linkage and absolute encoder.

The mobile robot consists of a 2D laser scanner, a landmark detector and a servo driver module. The intelligent lead output is used for generation of steering angle in modified VFH+ algorithm. The serial linkage is connected with rotary encoders or absolute encoders consecutively. The encoders which are selected should be small and precise. In general, however, larger encoders are more precise. MAE2-A2 made by USDigital Company is used in this study (Figure 3). The encoder is magnetic and an absolute encoder which provides PWM pulse with a 4,096 resolution at a 360° turn. Thus, 0.088° of accuracy per joint is found. In addition, the sampling rate of the PWM pulse is 250 Hz.

The Kinect sensor was attached to the end of the serial linkage to measure a user's shoulder and torso position. The Kinect sensor acquires depth information by applying IR patterns to an object and measuring them with an IR sensor. Compared to a general camera, there is no interference of ambient light, and it also works at night. Usability at night is considered an important characteristic for human-friendly guide robots.

We acquired a user's shoulder and torso position from depth image with OpenNI library. If the base of the serial linkage is shaken because a robot passes over a rough surface or makes a dynamic motion, the vibration is directly transmitted in the coordinates on a user's hand. To compensate for the vibration of the robot's base frame, an IMU is installed in the area which is held by the user's hand. Accelerometer and gyroscope data are used in the time prediction procedures. For this, an MTi sensor made by Xsens Company with 100 Hz of sampling rate has been used.

The three sensors mentioned above have measurement errors. First, in the case of the low-price IMU, the drift of the gyroscope causes cumulative errors. We used the encoder connected in the serial linkage with small products to reduce weight. As the size of an encoder decreases, accuracy declines as well. Also, as the serial linkage gets longer, the amount of position and pose errors increases proportionally. The position and pose errors differ depending on assembly accuracy as well as the length of the link and the encoder accuracy itself.

The Kinect sensor can suffer from occlusions by smoke/fog or obstacles. In addition, the performance of the Kinect sensor decreases in a dynamic environment. Therefore, it is necessary to reduce error in the integration system through fusion instead of separately using these sensors. This kind of integration can improve accuracy and prevent output errors generated from sensor failures from suddenly increasing. We made the serial linkage which consists of eight axes of rotation. We attached an absolute encoder to each axis. The link coordinate parameters of the serial linkage fabricated for the test is given in the table below (Table 1).

If ${\text{T}}_{\text{S}}^{8}$ is a transformation matrix from the IMU sensor coordinate frame to the last joint coordinate frame, the transformation matrix T from the IMU sensor coordinate frame to the base coordinate frame is calculated as follows:

(1) $$\text{T}={\text{T}}_{1}^{\text{B}}{\text{T}}_{2}^{1}{\text{T}}_{3}^{2}\cdots {\text{T}}_{8}^{7}{\text{T}}_{\text{S}}^{8}$$

If we denote the entry in the i-*th* row and the j-*th* column of the matrix T as *T*[*i,j*], the relative position and pose of sensor coordinates frame with respect to the navigation coordinate frame are calculated as follows:

(2) $$P_{x}=T\left[1,4\right],P_{y}=T\left[2,4\right],P_{z}=T\left[3,4\right]$$

(3) $$\theta =\tan^{-1}\left(\frac{-T\left[3,1\right]}{\sqrt{T{\left[3,2\right]}^{2}+T{\left[3,3\right]}^{2}}}\right),\psi =\tan^{-1}\left(\frac{T\left[2,1\right]}{T\left[1,1\right]}\right),\phi =\tan^{-1}\left(\frac{T\left[3,2\right]}{T\left[3,3\right]}\right)$$

In Equation (3), the notation *θ, ψ, φ* mean roll, pitch, yaw angle respectively.

## 3. Intelligent Lead Sensor

### 3.1. Serial Linkage/IMU Integration with EKF

The objective of using the serial linkage, the IMU and the Kinect sensor is to produce a stable distance from a user to a robot, speed-control volume and turn-control volume, even when the robot platform in which the base frame of the serial linkage is installed is shaken by a rough surface. To achieve this objective we acquire the hand position from the EKF with the serial linkage and the IMU.

**Figure 4.**
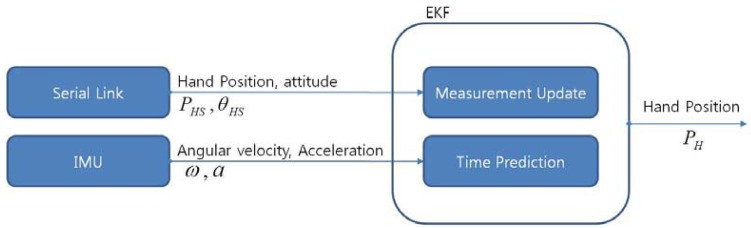
Block diagram of EKF for acquiring hand position.

In Figure 4, the symbol *P_HS_, θ_HS_* represent position data and orientation data of the IMU coordinate frame which is attached to the user's hand respectively. The symbols *ω* and *α* represent angular velocity and linear acceleration, respectively. The symbol *P_H_* means a position data calculated from the EKF. In this section, a system dynamic nonlinear equation, system matrix, and an output matrix which are necessary to design the EKF to fuse both the serial linkage and the IMU are introduced.

Table 2 represents the related symbols used to explain the EKF. The linear speed equation of the hand is as follows:

(4) $${\dot{p}}_{n}={\left(C_{n}^{b}\right)}^{T}v_{b}$$

where “*n*” refers to the definition in the navigation coordinate frame, and “*b*” refers to the definition in the body coordinate frame. The navigation frame is the same as the base frame of the serial linkage attached to the robot. The navigation coordinate frame is placed at the pinhole of the depth camera of the Kinect sensor. The body coordinate frame is located at the center of the IMU sensor. For the convenience of calculation, we assume that the hand coordinate frame coincides with the body coordinate frame. In the Equation (4)*v_b_* is defined in the body coordinate frame. However, a position term, *P_n_* is a data in the navigation coordinate frame at the composition of the system state which is defined below. Therefore, DCM (Direct Cosine Matrix) is multiplied.

The linear acceleration equation is as follows:

(5) $${\dot{v}}_{b}=\frac{1}{m}\;f_{b}+C_{n}^{b}g_{n}-\omega ⊗v_{b}$$

The quaternion equations of rotation velocity, which represent coordinate transformation from body coordinate frame to navigation coordinate frame, are expressed in a differential equation as shown below:

(6) $$\dot{q}=\frac{1}{2}q⊗\omega$$

The $C_{n}^{b}$ used in Equations (4) and (5) can be stated with the normalized quaternion as shown below:

(7) $$C_{n}^{b}=\left[\begin{array}{ccc} 1-2\left(q_{2}^{2}+q_{3}^{2}\right) & 2\left(q_{0}q_{3}+q_{1}q_{2}\right) & 2\left(-q_{0}q_{2}+q_{1}q_{3}\right)\\ 2(-q_{0}q_{3}+q_{1}q_{2}) & 1-2\left(q_{1}^{2}+q_{3}^{2}\right) & 2(q_{0}q_{1}+q_{2}q_{3})\\ 2\left(q_{0}q_{2}+q_{1}q_{3}\right) & 2\left(-q_{0}q_{1}+q_{2}q_{3}\right) & 1-2\left(q_{1}^{2}+q_{2}^{2}\right) \end{array}\right]$$

To induce state space representation from the nonlinear Equations (4∼6), state vector and output vector are defined as follows:

(8) $$\begin{array}{cc} x=\left[\begin{array}{c} p_{n}\\ v_{b}\\ q \end{array}\right], & y=\left[\begin{array}{c} p_{n}\\ v_{b}\\ \theta \end{array}\right] \end{array}$$

Then, the system matrix and the output matrix are obtained as follows:

(9) $$\begin{array}{cc} A=\left[\begin{array}{ccc} 0 & {\left(C_{n}^{b}\right)}^{T} & \frac{d\left(C_{b}^{n}v_{b}\right)}{dq}\\ 0 & -\omega ⊗ & \frac{d\left(C_{n}^{b}g_{n}\right)}{dq}\\ 0 & 0 & -\frac{1}{2}\omega ⊗ \end{array}\right], & H=\left[\begin{array}{ccc} 1 & 0 & 0\\ 0 & 1 & 0\\ 0 & 0 & \frac{d\theta }{dq} \end{array}\right] \end{array}$$

We need to first initialize *x_0_, P_0_, Q, R* and then to calculate time prediction procedure and measurement update procedure consequently. First we acquire roll, pitch and yaw angles from MTi sensor and then we convert Euler angle to quaternion *q_0_* with these data. The covariance matrix *P_n0_* is set to an initially calculated value from the serial linkage. And the initial value of *v_b_* is set to 0. From these values, the initial state and the initial covariance matrix are assigned as follows:

(10) $$x_{0}=\left[\begin{array}{c} p_{n0}\\ 0_{3\times 3}\\ q_{0} \end{array}\right],\;P_{0}=I_{10\times 10}$$

The process noise matrix and measurement noise matrix were determined to avoid the divergence of the filter output by trial and error as follows:

(11) $$Q=\left[\begin{array}{ccc} 0I_{3\times 3} & 0_{3\times 3} & 0_{3\times 4}\\ 0_{3\times 3} & 0.1I_{3\times 3} & 0_{3\times 4}\\ 0_{4\times 3} & 0_{4\times 3} & 0.00001I_{4\times 4} \end{array}\right]$$

(12) $$R=\left[\begin{array}{ccc} 0.8I_{3\times 3} & 0_{3\times 3} & 0_{3\times 4}\\ 0_{3\times 3} & 0.2I_{3\times 3} & 0_{3\times 4}\\ 0_{4\times 3} & 0_{4\times 3} & 1I_{4\times 4} \end{array}\right]$$

In the above Equation (12)*I_n_*_×_*_n_* represents *n* by *n* dimensional identity matrix.

The time prediction procedure shown in the block diagram above can be stated as follows:

1. With the current state vector and IMU sensor data, update the system matrix from the Equations (9).
2. Calculate the derivative of state vector by using the equation, *ẋ_n_*_−1_ = *Ax_n_*_−1_
3. Update the current state vector by using the equation, *x_n_* = *x_n_*_−1_ + *ẋ_n_*_−1_*dt*
4. Normalize the quaternion among the updated states.
5. Update the P matrix by using the equation, *P_n_* = *AP_n_*_−1_*A^T^* + *Q*

The measurement update procedure can also be stated as follows:

1. Determine the output matrix with the current quaternion from the Equation (9).
2. Measure the output, z*_m_* with the hand position, *P_HS_* and pose, *θ_HS_* measured from the serial linkage.
3. Calculate the output error by using the equation, *e* = *z_m_* − *Hx_n_*
4. Update Kalman gain by using the equation, *K* = *P_n_H^T^*[*HP_n_H^T^* + *R*]^−1^
5. Update the state vector by using the equation, *x_n_*_+1_ = *x_n_* + *Ke*
6. Update the P matrix by using the equation, *P_n_*_+1_ = *P_n_* − *KHP_n_*

The first 3-terms of the state vector, which are updated through the above procedures, are the coordinates of the hand position viewed from the base coordinate frame of the serial linkage. As mentioned previously, we assume that the base coordinate frame of the serial linkage is placed at the pinhole of the depth camera of the Kinect sensor.

### 3.2. Intelligent Lead Output Generation

The intelligent lead proposed in this study acquires a user's left shoulder position, right shoulder position and torso position from the Kinect sensor and hand position with Serial linkage and IMU on a real-time basis.

The intelligent lead has two buttons. One is the start button and another is the trim button. If the user pushes the trim button current output volumes are saved in trim buffers. The intelligent lead has a hand position fault checker block. If the distance from the shoulder to the hand is larger than the sum of the upper arm length and the lower arm length, than the output volumes become zero. This prevents unwanted generation of output volumes when the user loses hold of the handle. If the user's hand position is obtained from the output of the EKF, the user's control intention should be investigated by using this data and the user's right shoulder, left shoulder and torso position data which have been obtained from the Kinect sensor. Because the size of walking steps, the amount of back and forth arm swings and the amount of movements to the left and right differ from individual to individual, it is necessary to get information on changes of the user's arm-joints while walking.

**Figure 5.**
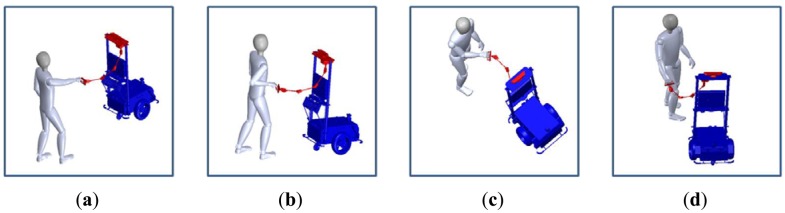
Control motions. (**a**) Speed Increase; (**b**) Speed Decrease; (**c**) Left turn; and (**d**) Right turn.

When people walk with a dog, it is more common to move the hand to the left and right to point out directions instead of changing the angle of the wrist. In addition, many people push their hand forward and pull down backward to increase or decrease the velocity. Figure 5 describes the actions for generating commands of speed increase, speed decrease, left turn and right turn respectively.

**Figure 6.**
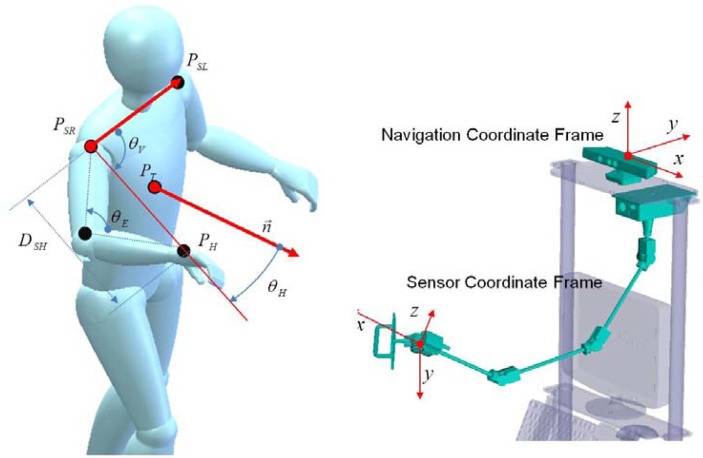
Frames, angles and distance definition.

We assume the user is a right-hander. Figure 6 shows frames, angles and distance defined from the joint positions.

The normal vector represents the direction of the plane in which a triangle is formed by the left shoulder position, the right shoulder position and the torso position. The symbol *D_SH_* means the size of the line created by the right shoulder position and the hand position. The symbol *θ_V_* means the angle between the normal vector and the line created by the right shoulder position and the hand position. The symbol *θ_H_* means the angle between the line created by the right shoulder position and the hand position and the line formed by the left shoulder position and the right shoulder position. The distance from the shoulder to the hand is calculated as follows:

(13) $$D_{SH}=\left\|P_{H}-P_{SR}\right\|$$

If the left shoulder position, *P_SL_* is (*x_1_y_1_z_1_*)*^T^*, right shoulder position, *P_SR_* is (*x_2_y_2_z_2_*)*^T^* and torso position, *P_T_* is*(x_3_y_3_z_3_)^T^*, then the normal vector *n⃗* of the plane that includes these three points can be calculated as follows:

(14) $$\vec{n}={\left(\begin{array}{ccc} \left|\begin{array}{cc} y_{2}-y_{1} & z_{2}-z_{1}\\ y_{3}-y_{1} & z_{3}-z_{1} \end{array}\right| & -\left|\begin{array}{cc} x_{2}-x_{1} & z_{2}-z_{1}\\ x_{3}-x_{1} & z_{3}-z_{1} \end{array}\right| & \left|\begin{array}{cc} x_{2}-x_{1} & y_{2}-y_{1}\\ x_{3}-x_{1} & y_{3}-y_{1} \end{array}\right| \end{array}\right)}^{T}$$

The angles defined above can be obtained using the law of cosine:

(15) $$\theta_{E}=\pi \pm \arccos\left(\frac{L_{UA}^{2}+L_{LA}^{2}-D_{SH}^{2}}{2L_{UA}\cdot L_{LA}}\right)$$

(16) $$\theta_{H}=\arccos\left(\frac{\left(P_{SL}-P_{SR})\cdot (P_{H}-P_{SR}\right)}{\left\|P_{SL}-P_{SR}\right\|\times \left\|P_{H}-P_{SR}\right\|}\right)$$

(17) $$\theta_{V}=\arccos\left(\frac{\vec{n}\cdot (P_{H}-P_{SR})}{\left\|\vec{n}\right\|\times \left\|P_{H}-P_{SR}\right\|}\right)$$

If the height of the hand position is above the height of the torso position, we assume that the user has the intention to control the robot's direction or speed. In addition, if *θ_V_* is above a certain limit value, then we conclude that the user bent his/her waist. We fixed the limited value to 45 degrees in the following test. In these cases the intelligent lead output forced to set 0. This assumption prevents unwanted output generation. The intelligent lead has the trim button to set trim values for speed control and turn control. The default trim value is as follows:

(18) $$\text{TRI}\;M_{\text{SPEED}}=\sqrt{L_{UA}^{2}+L_{LA}^{2}},\text{TRI}\;M_{\text{TURN}}=\frac{\pi }{2}$$

Finally we get intelligent lead output values for control command as follows:

(19) $$O_{S}=\{\begin{array}{cc} D_{SH}-\text{TRI}\;M_{\text{SPEED}} & ,if\;\theta_{V}<\frac{\pi }{4}\;\text{and}\;P_{H}\;(z)>P_{T}\;(z)\\ 0 & ,\text{otherwise} \end{array}$$

(20) $$O_{T}=\{\begin{array}{cc} \theta_{H}-\text{TRI}\;M_{\text{TURN}} & ,\text{if}\;\theta_{V}<\frac{\pi }{4}\;\text{and}\;P_{H}\;(z)>P_{T}\;(z)\\ 0 & ,\text{otherwise} \end{array}$$

In Equations (19) and (20), *O_S_* and *O_T_* mean speed control value and turn control value respectively, *P_H_*(*z*) and *P_T_*(*z*) represent z-axis value of hand and torso position respectively. The distance from the user's body to the robot can be calculated simply with the mean of two shoulder positions and a torso position:

(21) $$O_{D}=\frac{\left\|P_{SL}+P_{SR}+P_{T}\right\|}{3}$$

**Figure 7.**
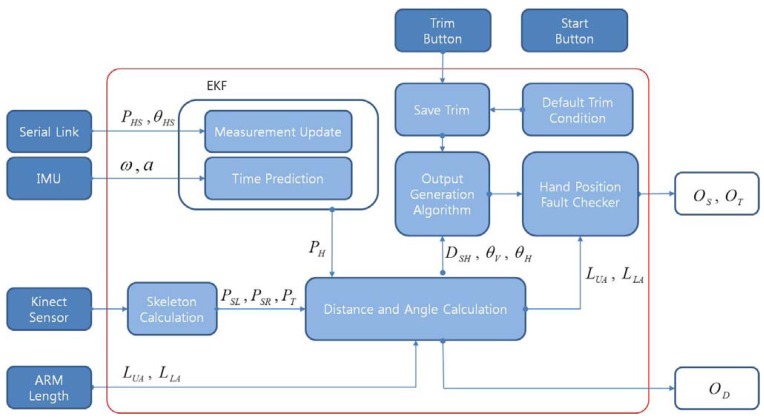
Block diagram of the intelligent lead.

The intelligent lead generates *O_D_, O_S_*, *O_T_* represented by the above Equations (19∼21) simultaneously on a real-time basis. Figure 7 shows the summary block diagram of the proposed intelligent lead.

Table 3 lists the related symbols for generating intelligent lead output.

## 4. Experimental Results

### 4.1. Mobile Robot for Testing

Many studies have proposed strategies for robots to complete their tasks without any collision with obstacles and/or other robots. Especially the methods using the potential field [13] and the Vector Field Histogram Plus (VFH+) algorithm [14,15] have given diverse and useful results. In this study, the robot direction and speed were controlled based on the VFH+ algorithm by using the obstacle information acquired from a 2D laser scanner and current location information acquired from an IR landmark detector. The size of the robot and local map were fixed to be 0.8 m and 3 × 3 meters in the VFH+ algorithm.

The HOKUYO UTM30-LX was used as a 2D laser scanner to detect obstacles. Even though UTM-30LX has measure range from −135° to 135°, the data measured from −90° to 90° was used in this test. Its specifications are listed in Table 4. Hagisonic's Stargazer was used as an IR landmark detector. We firmly affixed a total of 16 landmarks as IR reflective materials to the ceiling, whose height is 2.5 meters. Landmarks were aligned with a 1 meter interval horizontally.

To determine the steering angle with the VFH+ algorithm, it is first necessary to create a local map with 2D laser scanner data. Then from the local map, we obtain an obstacle vector, a Primary Polar Histogram, a Binary Polar Histogram, and a Masked Polar Histogram, in that order. Steering angle is calculated based on the final Masked Polar Histogram. The desired steering angle is simply determined by the sum of *h_vfh_* and *O_T_*, and steering angle error value *h_e_* is calculated by the difference desired angle and current angle as follows:

(22) $$h_{e}=\left(h_{\text{vfh}}+O_{T}\right)-h_{c}$$

In the above equation, *h_c_* is the current direction which is measured from the IR landmark detector and *O_T_* is the desired differential direction which is generated from the intelligent lead. The position error, *p_e_* is calculated as follows:

(23) $$p_{e}=\left(p_{d}+O_{s}\right)-O_{D}$$

**Figure 8.**
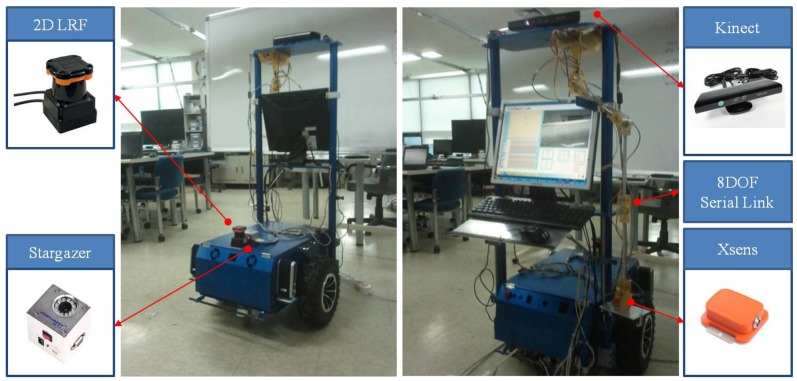
Experimental system.

In the above equation, *p_d_* represents the desired distance from a user to a robot which is fixed at 0.8 meters in this test. The symbol *O_S_* is the desired differential speed which is generated from the intelligent lead output, and *O_D_* is the measured distance from the user to the robot with the intelligent lead. For the test, a two wheel-type mobile robot was designed. Mitsubishi's 400W AC servo motor, 10:1 bevel gearbox, 13-inch wheels and PC (Intel i7-2600 CPU @3.4 GHz) were used. Figure 8 pictures the mobile robot and the intelligent lead sensor fabricated for the test.

**Figure 9.**
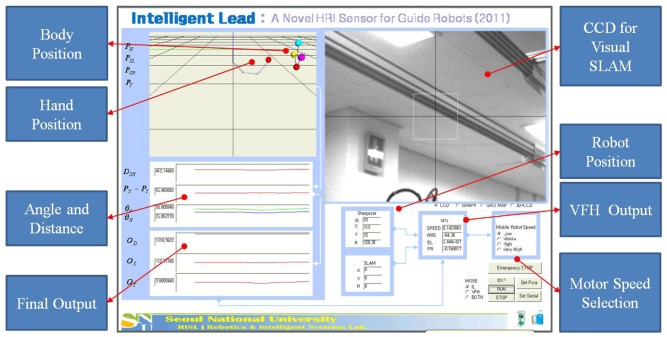
Monitoring program.

Figure 9 below shows the captured screen from the monitoring program made for our testing. We expressed calculated positions on the upper left side in 3D using the OpenGL. In Figure 9, the four dots on the top represent the user's head position, left shoulder position, right shoulder position, and torso position which have been detected by the Kinect sensor, respectively. In addition, the dot in the middle refers to the hand location which has been detected through the EKF. The values which were calculated from the four positions were marked on the bottom left. On the right side of the display a CCD camera image for visual tracking is shown, and on the bottom side we show current robot positions calculated from SLAM and detected from the Stargazer landmark detector. The values calculated from the VFH+ algorithm are displayed and selection buttons for servo motor speed setting are arrayed.

### 4.2. Test Results

**Figure 10.**
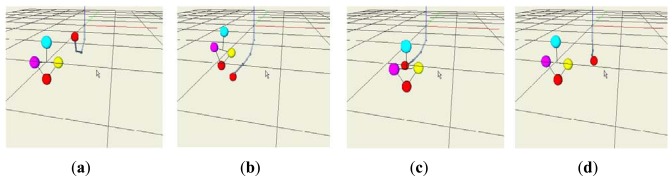
Measured positions. (**a**) Speed Increase; (**b**) Speed Decrease; (**c**) Left turn; and (**d**) Right turn.

In the first test, the control volumes were measured by moving the handle of the intelligent lead horizontally while the robot is stopped. Figure 10 shows acquired positions on the 3D viewer when we generated speed increase, speed decrease, left turn and right turn commands respectively.

**Figure 11.**
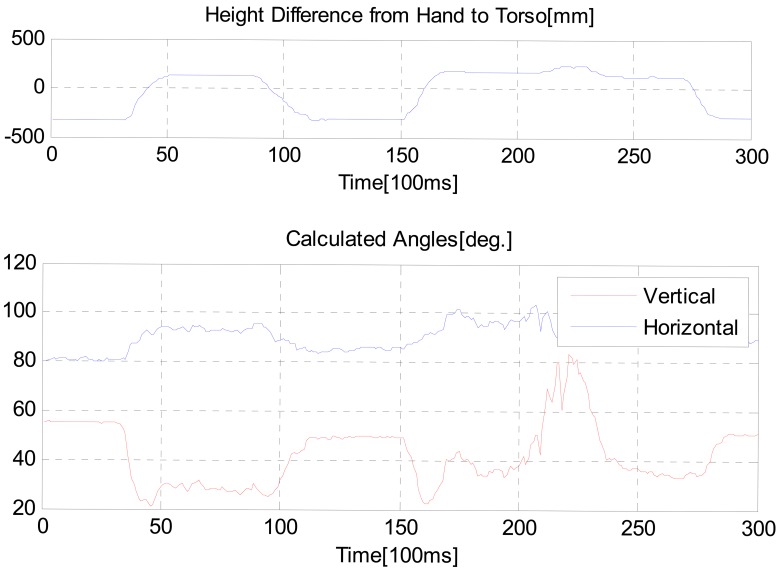
Calculated value.

Figure 11 shows measured values when the hand position was shaking. We intended that *θ_V_* changes via the defined limit value, 45 degrees and the height of the hand position goes from lower to upper than that of the torso position, and then from upper to lower.

**Figure 12.**
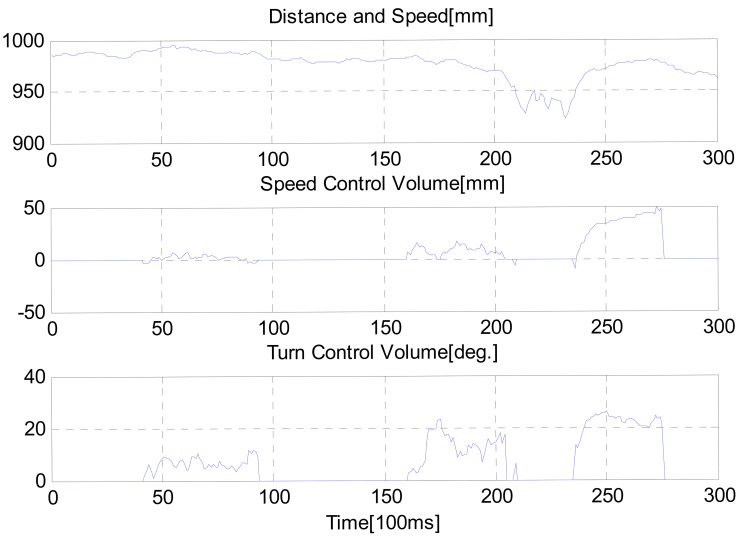
Sensor output.

As shown in the Figure 12, in the region of 200∼250 time tick, the user bent down at the waist. Since the height of the hand was above than that of the torso position, as *θ_V_* was larger than 45 degrees the intelligent lead output was not generated. The distance from the user to the robot was always generated, regardless of *θ_V_* value and the height of the hand position.

**Figure 13.**
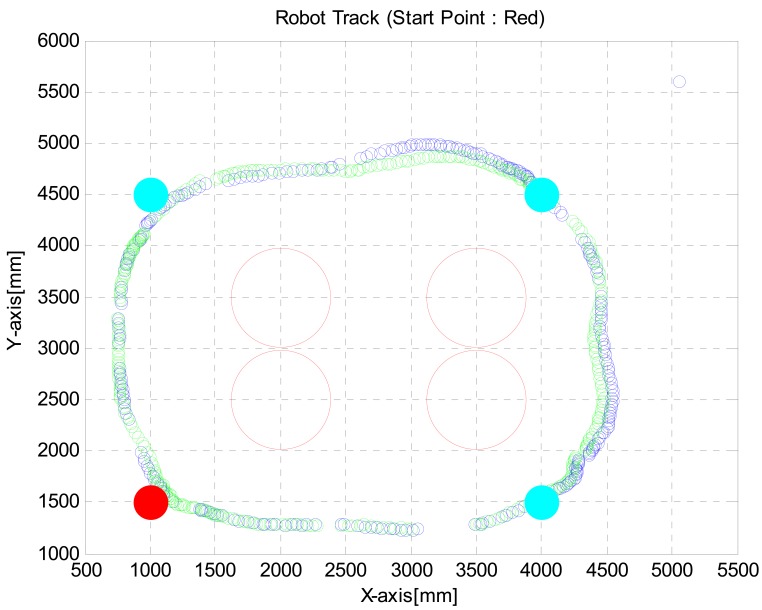
Robot track during VFH+ algorithm test.

In the second test, the robot was programmed to move to (1, 1.5), (1, 4.5), (4, 4.5) and (4, 1.5) points with avoiding obstacles twice by using VFH+ algorithm. We located obstacles at (2, 2.5), (2, 3.5), (3.5, 2.5) and (3.5, 3.5) points. The obstacles were cylinders with 0.5 meter diameter. Figure 13 shows robot track measured with the Stargazer. Because of the alignment and flatness errors of landmarks, it is shown that the robot path was cut off whenever detected landmarks are changing. Nevertheless the robot reached the desired destinations by avoiding obstacles.

**Figure 14.**
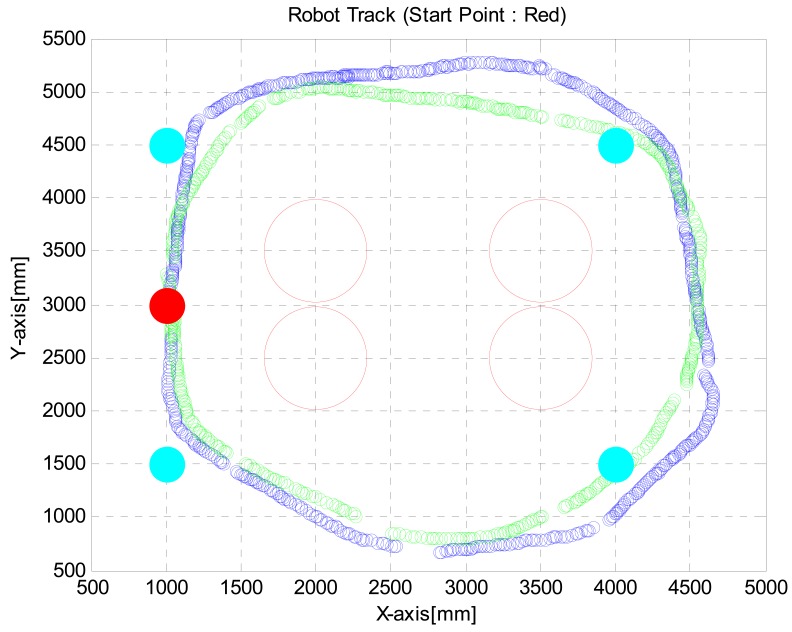
Robot track acquired from the third test.

When a robot is moving around following a user, it is difficult to use a joystick without a fixed joystick pad. The proposed intelligent lead can solve this problem. We show how to use intelligent lead as a joystick and a distance measure. In the third test, the ability of the robot equipped with the intelligent lead to reach a destination by avoiding obstacles was tested. The intention was for the robot to lead the users keeping 0.8 meter distance from the user. Our objective was to make a track twice by passing points at (1, 1.5), (1, 4.5), (4, 4.5) and (4, 1.5). We controlled the speed of the robot with the intelligent lead to make a first track within 120 seconds and then a second track within 80 seconds. And we generated turn control commands through the intelligent lead to arrive at the destinations and avoid obstacles. Figure 14 shows the robot track acquired from the third test. A red point means a start point and green points mean en route points or destination points, respectively. The result shows that we could reach the destination points keeping pre-defined distance from the robot.

**Figure 15.**
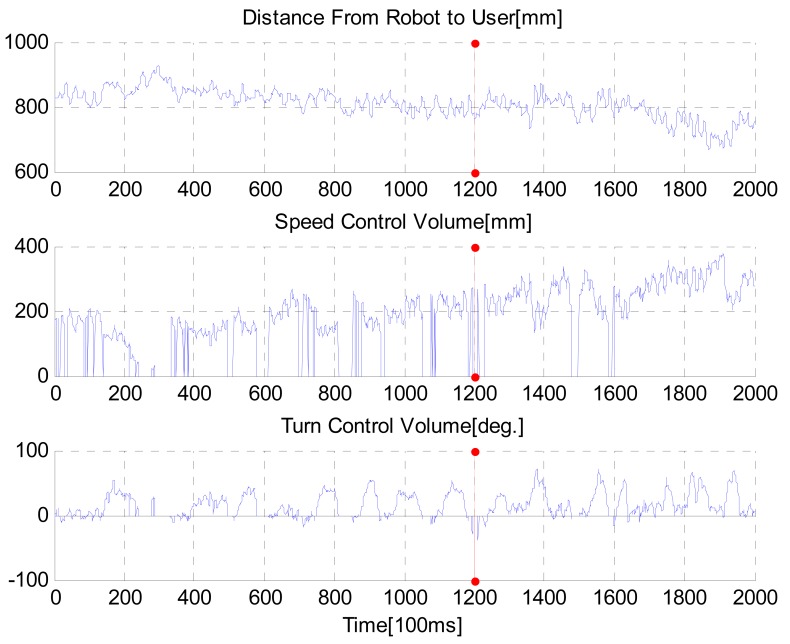
Sensor output acquired from the third test.

Output data from the intelligent lead during the test is shown in Figure 15. Mean distance between user and robot was 0.8101 meters. We confirmed that the proposed intelligent lead could be used as a new HRI joystick. Moreover, our results suggested that motion of humans could interact with robots using the proposed intelligent lead. During many tests, the OpenNI algorithm for searching skeleton fails occasionally at turning intervals. This is because the user gets out of the FOV due to the limited FOV. In future work, we need to convert the software to be able to solve this problem.

## 5. Conclusions

In this paper, we introduced the intelligent lead as a new HRI sensor. To detect the user's hand position, we made a serial linkage consisting of eight rotary encoders and then we constructed the intelligent lead with the Kinect sensor, the serial linkage and MTi sensor as an IMU. Their measurements were fused by an EKF. A mobile robot was designed to test the performance of the proposed intelligent lead and the related positions are monitored by calculating in 3D with the OpenGL. Several tests were conducted to verify that the mobile robot with the intelligent lead is capable of achieving its goal points while maintaining the appropriate distance between the robot and the user. It is shown that the intelligent lead acquires a stable distance from the user to the robot, speed-control volume and turn-control volume on a real-time basis. Moreover, our results suggested that the motion of humans could interact with the robot by the proposed intelligent lead in mobile environments such as those where the robot and the user are moving at the same time. During many tests, the OpenNI algorithm for searching skeleton failed occasionally at turning intervals for reasons of the limited FOV. In the future work, we need to convert the software to be able to solve this problem.

## Figures and Tables

**Table 1.** Link coordinates parameters of the serial linkage.

**Frame and Transformation Matrix**		***_θ_*_(deg.)_**	**_α__(deg.)_**	***_a_*_(mm)_**	***_d_*_(mm)_**
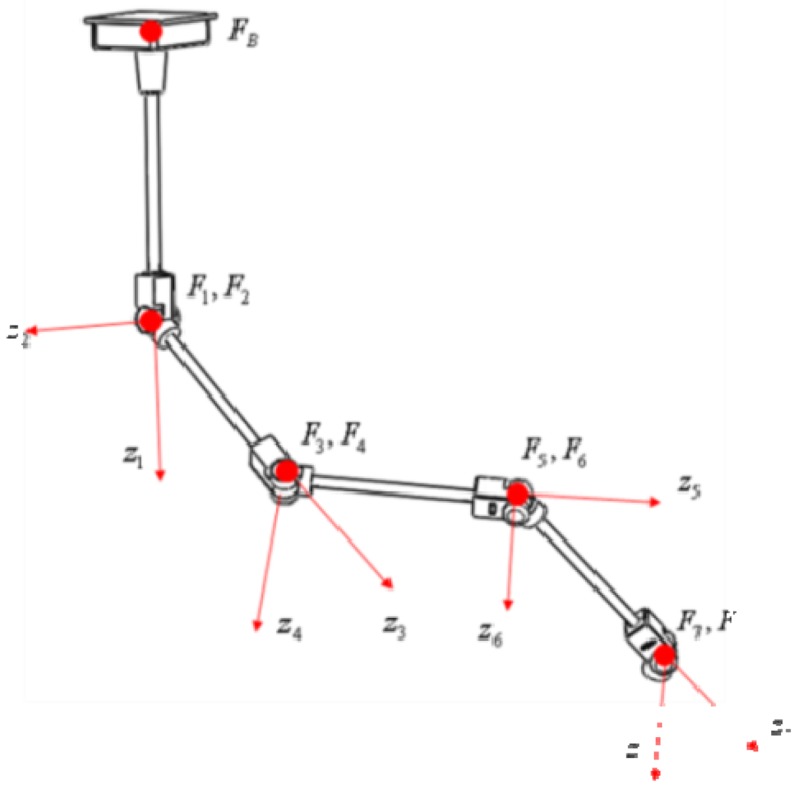	$T_{1}^{B}$	*_θ_*_1_	0	0	50
	$T_{2}^{1}$	*θ*_2_	90	0	0
	$T_{3}^{2}$	*θ*_3_	−90	0	200
	$T_{4}^{3}$	*θ*_4_	90	0	0
	$T_{5}^{4}$	*θ*_5_	−90	0	200
	$T_{6}^{5}$	*θ*_6_	90	0	0
	$T_{7}^{6}$	*θ*_7_	−90	0	200
	$T_{8}^{7}$	*θ*_8_	90	0	0
	$T_{\text{R}}^{8}$	0	−90	0	10

**Table 2.** Symbols used for EKF.

**Symbol**	**Meaning**	**Symbol**	**Meaning**
*P_n_*	Hand position	*Q*	Process noise matrix
$C_{b}^{n}$	Direct Cosine Matrix	*R*	Measurement noise matrix
*v_b_*	Velocity	*x*	State Vector
*m*	Mass	*y*	Output Vector
*f_b_*	Force	*θ*	Euler angle
*g_n_*	Gravity	*A*	System Matrix
*q*	Quaternion	*H*	Output Matrix
*P*	Covariance matrix	*K*	Kalman gain

**Table 3.** Symbols for intelligent lead output generation.

**Symbol**	**Meaning**	**Symbol**	**Meaning**
*P_H_*	Hand Position calculated from EKF	*O_S_*	Speed Control Volume
*P_SL_*	Left Shoulder Position	*O_T_*	Turn Control Volume
*P_SR_*	Right Shoulder Position	*O_D_*	Distance Output from Body to Robot
*P_T_*	Torso Position	*D_SH_*	Distance from Shoulder to Hand
*θ_E_*	Angle of Elbow	*n⃗*	Normal vector generated Body plane
*θ_V_*	Angle of Vertical	*L_UA_*	Length of Upper Arm
*θ_H_*	Angle of Horizontal	*L_LA_*	Length of Lower Arm

**Table 4.** Hokuyo UTM-30LX specifications.

	**Parameter**	**Typical value**
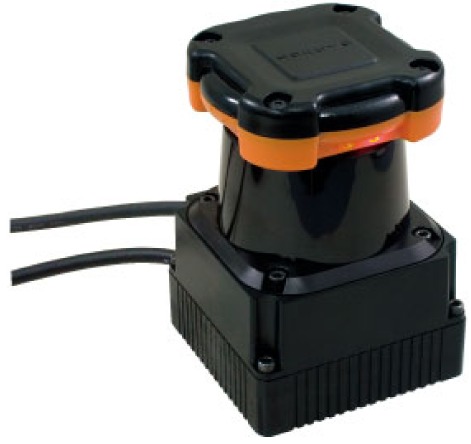	Light Source	Laser diode (λ = 785 nm), Class 1 (FDA)
	Detection Range	0.1 to 30 m, Max. 60 m
	Accuracy	0.1 to 10 m: ±30 mm, 10 to 30 m: ±50 mm
	Scan Angle	270°
	Angular Resolution	0.25° (360°/1,440 steps)
	Scan Time	25 msec/scan
	Interface	USB2.0 (Full Speed)
